# Discrete Redox Signaling Pathways Regulate Photosynthetic Light-Harvesting and Chloroplast Gene Transcription

**DOI:** 10.1371/journal.pone.0026372

**Published:** 2011-10-19

**Authors:** John F. Allen, Stefano Santabarbara, Carol A. Allen, Sujith Puthiyaveetil

**Affiliations:** 1 School of Biological and Chemical Sciences, Queen Mary University of London, London, United Kingdom; 2 Istituto di Biofisica, Consiglio Nazionale delle Ricerche, Milano, Italy; University of Melbourne, Australia

## Abstract

In photosynthesis in chloroplasts, two related regulatory processes balance the actions of photosystems I and II. These processes are short-term, post-translational redistribution of light-harvesting capacity, and long-term adjustment of photosystem stoichiometry initiated by control of chloroplast DNA transcription. Both responses are initiated by changes in the redox state of the electron carrier, plastoquinone, which connects the two photosystems. Chloroplast Sensor Kinase (CSK) is a regulator of transcription of chloroplast genes for reaction centres of the two photosystems, and a sensor of plastoquinone redox state. We asked whether CSK is also involved in regulation of absorbed light energy distribution by phosphorylation of light-harvesting complex II (LHC II). Chloroplast thylakoid membranes isolated from a CSK T-DNA insertion mutant and from wild-type *Arabidopsis thaliana* exhibit similar light- and redox-induced ^32^P-labelling of LHC II and changes in 77 K chlorophyll fluorescence emission spectra, while room-temperature chlorophyll fluorescence emission transients from *Arabidopsis* leaves are perturbed by inactivation of CSK. The results indicate indirect, pleiotropic effects of reaction centre gene transcription on regulation of photosynthetic light-harvesting *in vivo*. A single, direct redox signal is transmitted separately to discrete transcriptional and post-translational branches of an integrated cytoplasmic regulatory system.

## Introduction

Chloroplasts of photosynthetic eukaryotes are cytoplasmic organelles that have evolved from endosymbiotic, prokaryotic, cyanobacteria [1], [2], [3], [4]. Chloroplasts and cyanobacteria share the unique property of oxygenic photosynthesis, in which water serves as the electron donor to a chain of electron carriers that includes two separate energy-transducing photochemical reaction centres, each bound to a distinct population of light-harvesting pigments. A reaction centre together with its light-harvesting system and primary electron donors and acceptors comprises a photosystem [5]. The two distinct photosytems of chloroplasts and cyanobacteria, termed photosystem I and photosystem II, are connected in series in the photosynthetic electron transport chain [6], and their rates of electron transport are therefore equal for linear, or non-cyclic, electron transport.

The differing composition of light-harvesting pigments confers different absorption and action spectra on photosystem I and photosystem II [7]. The spectral composition of incident light changes in natural environments. The requirement for sustained and effective conversion of absorbed light energy by two reaction centres turning over at equal rates is satisfied by regulatory processes that adjust (a) the relative quantities of the two photosystems [8], [9] and (b) their light-harvesting antenna size and composition [10], [11], [12], [13]. Both of these adjustments compensate for an initial, transient imbalance in rate of reaction centre turnover that causes changes in the redox state of one or more electron carriers located between photosystem I and photosystem II. Since photosystem II donates electrons to photosystem I, their connecting electron carrier, plastoquinone, becomes chemically reduced when photosystem II is favoured by a new light regime, and oxidised when photosystem I is favoured, instead.

In chloroplasts, post-translational modification by phosphorylation of apoproteins of chloroplast light-harvesting complex II (LHC II) [14], [15] is initiated when plastoquinone becomes reduced [16], [17], [18], [19], [20], [21]. Phosphorylation of LHC II causes a sub-population of LHC II molecules to move from photosystem II to photosystem I [22], [23], redistributing absorbed excitation energy so that the rates of photochemical conversion in the two reaction centres are made equal. Thus a predominance of the reduced form of plastoquinone, plastoquinol, initiates a self-correcting response, a transition to a state of adaptation to light otherwise absorbed primarily by photosystem II [16], [17], [24], [25], [26]. This adaptation to a “light 2” is known as a transition to “light-state 2” or just “state 2” [7], [13], [24], [25], [27], [28]. Conversely, transient oxidation of plastoquinone inactivates phosphorylation of LHC II, and a light- and redox-independent phospho-LHC II phosphatase [18], [29] acts to return the mobile subpopulation of LHC II from photosystem I to photosystem II in an adaptation to “light 1” termed the transition to a “light-1 state” or “state 1” [13], [30].

The protein kinase catalysing phosphorylation of LHC II in a redox-dependent reaction, initiating the state 2 transition, has been identified as stt7 in *Chlamydomonas* [31], homologous with stn7 in *Arabidopsis* [30], [32], [33], [34], [35]. The phospho-LHC II phosphatase required for the state 1 transition has been identified as PPH1 [36] also termed TAP38 [37], [38].

As distinct from state transitions, changes in the quantity of photosystem I relative to that of photosytem II are also induced by changes in spectral composition of light absorbed and converted in photosynthesis [8], [9], [39]. These changes in photosystem stoichiometry represent an adaptation, or acclimation, that is complementary to state transitions, achieving balanced operation of photosystem I and photosystem II. While state transitions are a relatively rapid, reversible, post-translational solution to changing spectral composition, photosystem stoichiometry adjustment is a more long-term acclimatory response, taking hours or days to complete, and involving control of gene expression at the level of transcription and/or translation [9], [39], [40]. State transitions are superimposed on different photosystem stoichiometries and occur apparently independently of the ratio of photosystem I to II [39], [41], although the variable chlorophyll fluorescence often used to monitor state transitions *in vivo* is influenced by both reaction centre stoichiometry and light-harvesting antenna size [42]. A major factor affecting fluorescence yield is the antenna size of photosystem II, since this is the origin of the variable component of chlorophyll fluorescence at room temperature.

Photosystem stoichiometry adjustment has been shown to be initiated, like state transitions, by changes in redox state of plastoquinone [43], [44], [45]. Thus a prolonged light 2 alters gene expression and results in an increase in the stoichiometry of photosystem I to photosystem II. In plants, this change may be monitored easily as an increase in the ratio of chlorophyll *a* to chlorophyll *b*. The core apoproteins of the photosystem I and II reaction centres are the products of genes in chloroplast DNA. Studies of transcription in isolated chloroplasts demonstrated that photosystem I transcription is induced, while photosystem II transcription is repressed, upon reduction of plastoquinone. Conversely, photosystem I is repressed, and photosystem II induced, upon oxidation of plastoquinone. These experiments [43], [44], [45] introduced the possibility of studying early events in control of photosystem stoichiometry in vitro.

A conserved redox sensor kinase, Chloroplast Sensor Kinase (CSK), has been shown to be required for the plastoquinone redox-state dependent regulation of chloroplast reaction centre gene transcription [46], [47]. *Arabidopsis* knockout mutants of the *CSK* gene are unable to repress photosystem I genes in light absorbed predominantly by photosystem I (“light 1”), and therefore cannot regulate the stoichiometry of photosystem I relative to photosystem II [47]. CSK is a bacterial-type sensor kinase that belongs to the family of two-component signalling proteins [48], [49]. CSK has homologues in all major lineages of photosynthetic eukaryotes [46], [50]. In the complete genome sequences of the chlorophycean alga *Chlamydomonas reinhardii* and the haptophyte *Emiliania huxleyi*, however, no *CSK* gene is identified by similarity searches. Nevertheless, the possibility exists that the histidine kinase-like chlamyopsin protein replaces CSK in *Chlamydomonas* and that the plastid-encoded histidine kinase ycf26 compensates for the lack of CSK in *Emiliania* [50]. A CSK homologue is also found in cyanobacteria, suggesting the cyanobacterial ancestry of this chloroplast protein. The functional partner of CSK in plants and green algae [46] is not a response regulator as in canonical bacterial two-component systems, but a eukaryotic serine/threonine protein kinase known as Plastid Transcription Kinase (PTK) [51], [52] and a chloroplast sigma factor, SIG1 [53], [54]. Reversible phosphorylation of SIG1 by CSK is thought to be the mechanism by which photosystem I genes are repressed in light 1 condition, as part of the photosystem stoichiometry adjustment [46].

Given that the same changes in plastoquinone redox state initiate both light-state transitions and complementary changes in chloroplast reaction centre gene transcription [40], [55], it is of interest to ask whether Chloroplast Sensor Kinase is required for state transitions. Light-induced changes in the rate of run-on chloroplast transcription can be observed in as little as fifteen minutes, suggesting the possibility of synchronous induction of state transitions and the transcription control that leads, on longer time scales, to changes in photosystem stoichiometry [40].

Here we report on state transitions in wild-type *Arabidopsis thaliana* and in a CSK T-DNA insertion line. State transitions were monitored as one of several components affecting room-temperature chlorophyll fluorescence yield *in vivo*, and by 77 K fluorescence spectroscopy of isolated chloroplast thylakoids. In addition, direct visualisation of thylakoid protein phosphorylation was carried out by autoradiography of protein gels from samples incubated with [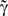 ^32^P]ATP.

## Results

**Figure 1:**
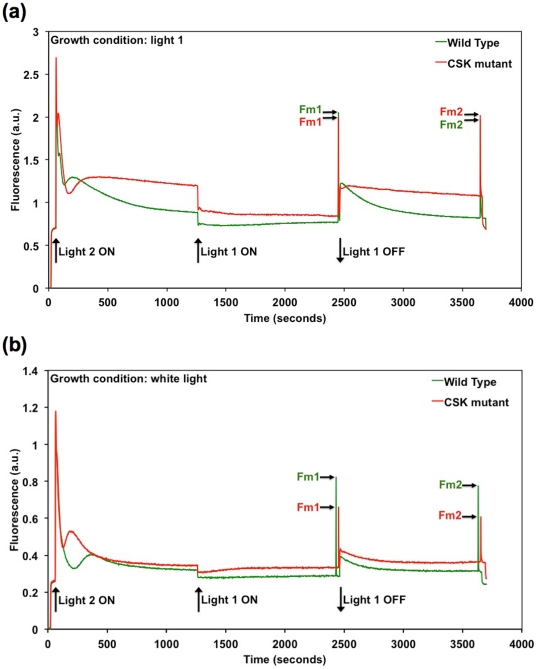
Chlorophyll fluorescence emission by leaves of *Arabidopsis thaliana* at room temperature. Two days of light 1 treatment (before monitoring the room temperature variable chlorophyll fluorescence) are assiciated with an apparent state transition minus phenotype in CSK mutants. Figure 1a shows the time-course of variable chlorophyll fluorescence emission from leaves of *Arabidopsis thaliana*, which were grown in light 1. Illumination with light 2, which is absorbed primarily by photosystem II, initially increases chlorophyll fluorescence emission. Fluorescence then decreases, and one component of the decrease is the removal of light-harvesting capacity from photosystem II during the transition to state 2. Addition of light 1, absorbed primarily by photosystem I, causes an initial decrease in fluorescence and then a slow rise, as the light-harvesting capacity of photosystem II increases during the transition to state 1. The slow components attributable to the state 2 and state 1 transitions are seen in the wild type, but are absent from the CSK mutant. Figure 1b shows fluorescence emission from white light grown plants. Fm 1 and Fm 2 are maximal fluorescence at state 1 and state 2 respectively.

Figure 1 shows the effects of actinic light 1 and light 2 on room-temperature chlorophyll fluorescence yield of one leaf, selected for size and thus signal amplitude, of *Arabidopsis thaliana* plants growing on compost. Figure 1 (a) shows results obtained with plants pre-adapted for 48 hours to growth in a red and far-red enriched light 1; figure 1 (b) shows results obtained with plants maintained in normal, low-irradiance, white light from fluorescent strips.

Measurements of the amplitude of chlorophyll fluorescence emission, in dark adapted leaves and in presence of continuous background lights, provide information on photochemical and non-photochemical quenching, with components also arising from high-energy state quenching [56] as well as state 1-state 2 transitions [57]. In dark-adapted leaves, the maximal photochemical yield of PS II, as determined from the Fv/Fm ratio, is essentially the same in the wild-type and the CSK mutant, and has the value of 0.80. Moreover, this value is unchanged by the growth conditions, both in the wild-type and the CSK mutant. This indicates that inactivation of the *CSK* gene does not affect the maximal photochemical yield of PS II.

However, upon the onset of background actinic illumination which excites preferentially PS II (light 2), difference can be observed, both in the pre-steady state kinetics manifested in the so-called “Kautsky” effect and the steady-state level of fluorescence emission between the wild-type and the CSK mutants (Figure 1). Particularly, in plants grown under light 1 conditions (Figure 1a), the onset of the Kautsky transient, which is an indication of the activation of Calvin circle, is delayed in the CSK mutant with respect to the wild-type, moreover the steady-state fluorescence emission level in the CSK mutant is significantly higher than in the wild-type. This is an indication that, under the same intensity and spectral distribution of actinic illumination, the plastoquinone pool is more reduced in the CSK mutant than in the wild-type control.

Superimposition of a light that preferentially excites PS I (light 1) after 20 minutes causes a drop in fluorescence emission. This is commonly explained in terms of an oxidation of the plastoquinone pool driven by PS I photochemistry which is in part limited by photon absorption when light 2 acts as the actinic source. The initial effect of light 1 superimposition is accentuated in the CSK mutant, which might indicate that the high reduction level of PQ pool observed under light 2 actinic illumination results only from PS I limitation of the linear electron transport chain of thylakoids. Moreover, while in the wild type there is a slow fluorescence rise after light 1 superimposition, in the case of CSK mutant the steady-state level continues to drop slowly. The slow rise in fluorescence observed in the wild type was attributed both in part to oxidation of a fraction of PQ pool, in part to redistribution of LHC antenna, increasing the cross section of photosystem II during the transition to state 1. Thus the difference observed in the CSK mutant might be indicative of a perturbation of state 1-state 2 transitions. However, influence of onset and relaxation of the high-energy component of non-photochemical quenching would also contribute to the steady state signal. Yet, we notice that the maximal fluorescence levels determined with a brief saturating pulse (∼800 ms) immediately before the actinic light 1 is switched off (Fm1 in Figure 1a) are very similar in the CSK and the wild type. This indicates that, for plants adapted to light 1 conditions, the level of Non Photochemical Quenching (NPQ) and the absorption cross section in the CSK mutant and the wild-type do not differ significantly, as the probability that these two processes compensate exactly for each other is extremely low. However, while the value of the Fm level in the presence of actinic light 2 (Fm2) is slightly lower (∼15%) than Fm1 for the WT these two levels are virtually identical for the CSK mutant. As the changes observed in the WT are consistent with the previous estimate of the mobile LHC during state transitions, this might indicate some impairment of state transition in the CSK deficient mutant. We note that, when light 1 is turned off, the steady state reduction level of PQ pool is again much higher in the CSK mutant compared to the WT. This indicates that the elevated emission under actinic illumination in the CSK mutant, also observed during the first period of light 2 illumination, is not the result of pre-steady state conditions and originates from a more reduced PQ pool in the mutant than in the wild-type.

Interestingly, some significant difference between plants grown under standard white light conditions (Figure 1b) and plants adapted to light 1 (Figure 1a) are apparent. Excluding the Fv/Fm values, which are essentially the same, it can be seen (Figure 1b) that: (i) the steady-state levels of fluorescence emission in presence of actinic backgrounds are much more similar in the CSK mutant and WT; (ii) the pre-steady state kinetics of the Kautsky transient are faster in CSK mutant than in the WT while the opposite was seen for light 1 adaptation; (iii) we observe a significant increase in the level of non-photochemical quenching in CSK mutants with respect to WT, whereas similar levels were observed in light 1 adapted leaves; (iv) the Fm1 level is greater than Fm2 in both wild-type and CSK mutants (Figure 1b). The increase in non-photochemical quenching can explain in part the difference in the steady-state level of emission observed in CSK mutant adapted to white light or light 1 conditions, as it will tend to lower the fluorescence emission even in the presence of a more reduced PQ pool, which was more clearly observed in light 1-adapted plants, but it is also apparent when actinic light 1 and light 2 are superimposed, and during the second period of light 2 illumination only (Figure 1b). Moreover the apparent rapid kinetic relaxation of the Kautsky transient in the CSK mutant could also be in part due to the onset of NPQ rather than a more rapid attainment of steady-state level. We are unaware of protein kinases controlling any of the non-photochemical quenching component, however this result (Figure 1b) can be ascribed to an indirect effect, linked to an inability of the CSK mutant to regulate the synthesis of photosynthetic complexes in response to changes in environmental conditions [47].

**Figure 2:**
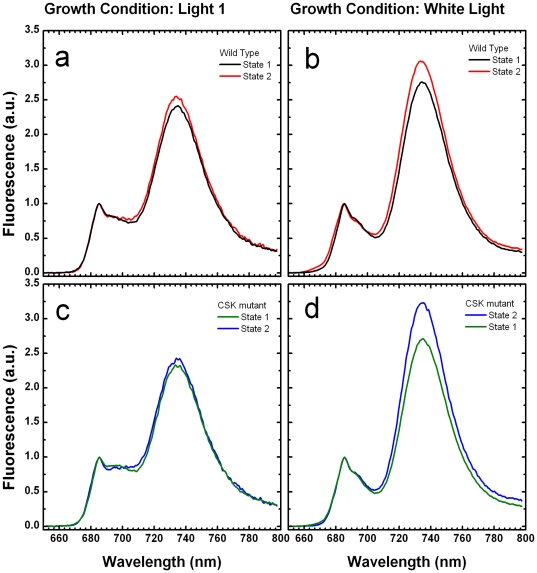
Fluorescence emission spectra of isolated thylakoids at 77 K. 77 K fluorescence emission spectra from wild type and CSK null mutant thylakoids as measured by the Perkin-Elmer LS 55 luminescence spectrometer. Figure 2a and 2c shows fluorescence emission spectra of thylakoids isolated from light 1 adapted plants. Figure 2b and 2d show fluorescence emission spectra of thylakoids isolated from white light grown plants. The excitation wavelength was 435 nm (5 nm slit width) and emission was detected from 650 to 800 nm (2.5 nm slit width). All spectra were normalized at 685 nm.

As a complete interpretation of the steady-state fluorescence emission in presence of actinic background is complicated by the presence of many different physiological processes, we investigated the effect of CSK in the control of state-transitions by fluorescence emission spectroscopy at 77 K of isolated thylakoids. Spectra are shown in Figure 2. In all cases, two principal fluorescence emission maxima are seen, one centred at 685 nm, also associated with a shoulder at ∼700 nm, and arising principally from photosystem II emission and the other, centred at 735 nm, which originates from photosystem I. The intensity of the 685 nm and 735 nm peaks observed at 77 K can be used to estimate the *relative* absorption cross-section of the two photosystems, and hence on state transitions. The state 2 transition was induced *in vitro* by illumination of thylakoids in the presence of ATP, and state 1 was produced from thylakoids incubated in the dark with ATP. As a control, thylakoids were incubated in the dark without ATP, and this treatment also induces state 1 (results not shown). It is seen in all cases that the emission ratio F735/F685 is greater in state 2 than in state 1, most noticeably in thylakoids from white light-grown plants (Figure 2b and 2d). However, the effect of the ATP and illumination on excitation energy distribution between photosystems I and II is much the same in the CSK mutant (Figure 2c, 2d) as in the wild-type (Figures 2a, 2b). Thus, neither the Fm values from white light grown plants at room temperature (Figure 1b) nor 77 K fluorescence emission spectra (Figure 2) indicate any effect of the CSK mutation on redistribution of excitation energy in light-state transitions.

**Figure 3:**
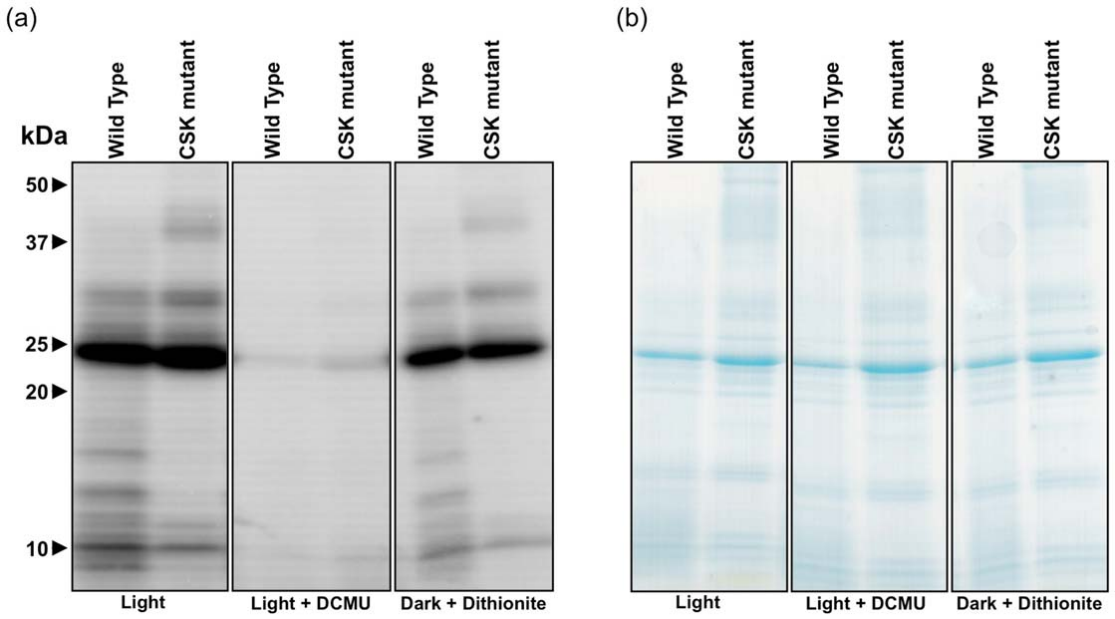
CSK mutants show normal LHC II phosphorylation. (a) Autoradiographs of *Arabidopsis* thylakoid phosphoproteins separated by SDS-PAGE. The positions of molecular weight markers are indicated on the left. Thylakoid samples from the wild-type (WT) and the CSK mutant are loaded in each lane and labelled accordingly at the top. The experimental conditions for each pair of samples are labelled at the bottom. (b) Coomassie-stained gel as protein loading control. The gel from which the autoradiograph was developed is stained with Coomassie brilliant blue to show that results presented in (a) results from ^32^P-labelling and not from unequal protein loading.

Phosphorylation of light harvesting complex II (LHC II) by the LHC II kinase induces state 2 transition, and its dephosphorylation by the phospho-LHC II phosphatase leads to state 1 transition [16], [25]. In order to further probe the role of CSK in state transitions, we carried out a thylakoid phosphorylation assay for the CSK mutant and the wild-type. Thylakoids were first isolated from white light grown plants, and incubated in light for 10 minutes in the presence of [*γ*-^32^P] ATP. The results of ^32^P-labelling experiments are shown in Figure 3. Incubation of thylakoids in white light induces the state 2 transition via phosphorylation of LHC II. Figure 3 shows equal levels of LHC II (protein band around 25 kDa) phosphorylation in both CSK mutant and wild type. In the light, the electron transport inhibitor 3-(3,4-dichlorophenyl)-1,1-dimethylurea (DCMU) inhibits electron transport to plastoquinone and makes the plastoquinone pool oxidised. Oxidised plastoquinone promotes the state 1 transition, and LHC II remains in an unphosphorylated state. Thus, incubation of wild-type and CSK mutant thylakoids with DCMU in the presence of light abolishes ^32^P-labelling of LHC II (Figure 3).

Dark-incubated thylakoids are in state 1, as the plastoquinone pool is usually oxidised in the dark, but state 2 can be induced in dark-incubated thyalkoids by the addition of the reducing agent sodium dithionite. Incubation of thylakoids in the dark in the presence of sodium dithionite results in phosphorylation of LHC II, more or less equally in both CSK mutant and wild-type (Figure 3a). In addition to LHC II, other thylakoid proteins are known to become phosphorylated in response to changes in plastoquinone redox state, and the phosphorylation pattern of several additional polypeptides appears to be different in CSK mutant and wild-type (Figure 3a). In particular, phosphorylation of photosystem II reaction centre proteins D1 and D2, at 32–34 kDa, is more pronounced in the CSK mutant. Reaction centre phosphoryation is not dorectly related to state transitions. Phosphorylation of polypeptides at 10 to 20 kDa is also affected in the CSK mutant (Figure 3a). Figure 3b reports substantially similar thylakoid polypepetide composition between the CSK mutant and wild-type, except in the upper part of the gel where some of the higher molecular weight polypeptides are unresolved in the CSK mutant. It is possible that these polypeptides are present, but aggregated near the top of the gel. However, these changes cannot account for the large redox-dependent effects on LHC II phosphorylation (Figure 3a) and may arise from effects of CSK on photosystem stoichiometry and from further, pleiotropic effects, as yet uncharacterized.

## Discussion

Chloroplast Sensor Kinase provides the redox-signalling machinery that connects plastoquinone redox state to chloroplast reaction centre gene transcription during photosystem stoichiometry adjustment [47], [58]. Changes in plastoquinone redox state also trigger light state transitions, and CSK could act as the common plastoquinone sensor in both stoichiometry adjustment and state transitions. However, the data presented in figures 2 and 3 argue against direct involvement of CSK in state transitions. Room temperature chlorophyll fluorescence data for light 1-acclimated plants (Figure 1a) indicate that *CSK* gene inactivation causes pleiotropic effects. Two days of light 1 acclimation before measuring state trasitions is considered to increase photosystem II antennae, and therefore light 1-acclimated plants are expected to undergo robust state transitions. It is not clear what caused this pleiotropic effect on room temperature fluorescence in CSK mutants in light 1 (Figure 1a). It should be noted that unlike the earlier study where two independent T-DNA lines have been analysed for the gene expression phenotype of CSK [47], the present study employed only one of the two CSK T-DNA lines. The chances of the room temperature phenotype (Figure 1a) arising from a secondary mutation other than the *CSK* gene locus, although unlikely, cannot be completely excluded. Since CSK is known to regulate stoichiometry of photosystems, it may be that light-1 acclimated CSK mutants are unable to adjust relative quantities of the two photosystems, and that this altered stoichiometry manifests itself as an aberrant room temperature fluorescence property. But given the independent nature of state transitions and photosystem stiochiometry adjustments [59] this possibility seems less certain. Other factors such as altered photochemical quenching, high-energy quenching or LHC II gene (*cab* or *Lhcb*) expression may also account for this anomaly in CSK mutants. It is also important to consider that state transition, Stn7 mutation may, conversely, have indirect, pleiotropic effects on reaction centre gene transcription [34] and thus on photosystem stoichiometry.

**Figure 4:**
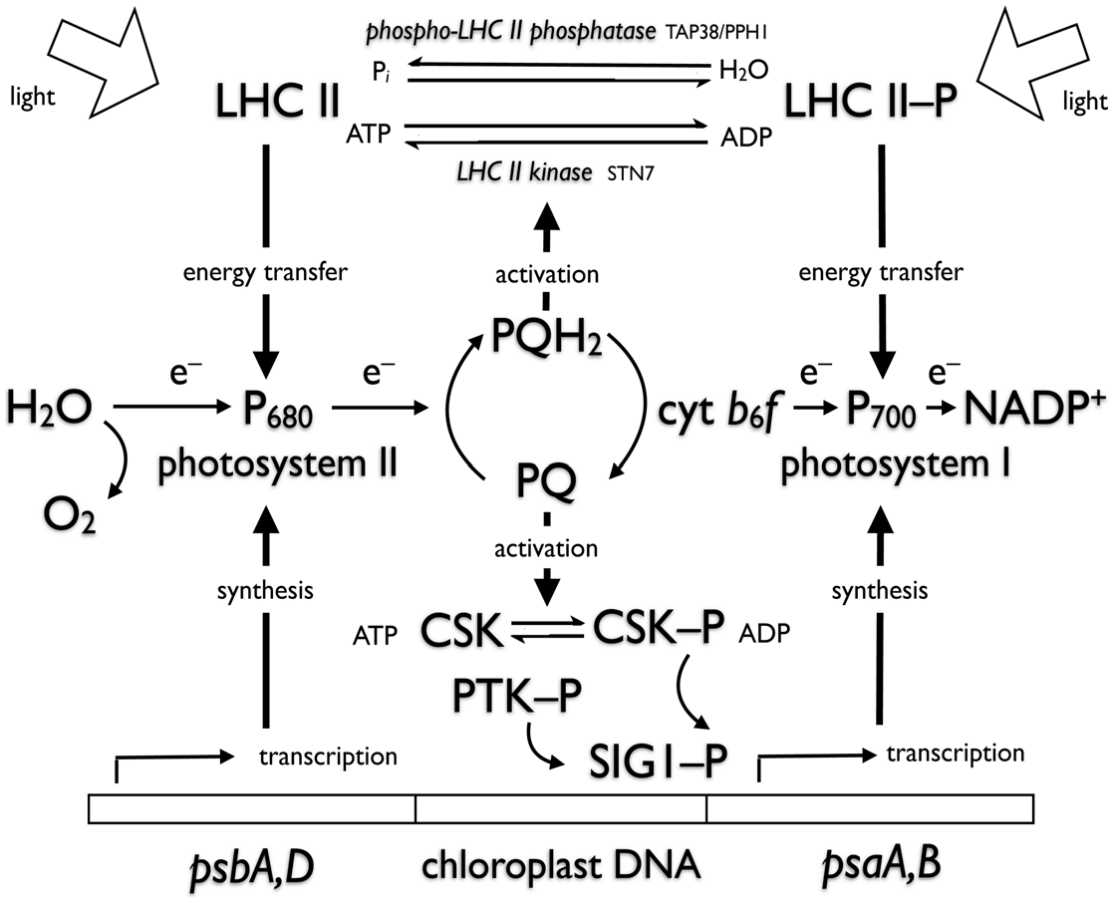
Interactions of photosynthetic electron carriers with redox-signalling components of photosystem stoichiometry adjustment and state transitions. Light reactions of photosynthesis are represented as electron transport from H_2_O to NADP^+^ via two photosystems connected by a cytochrome *b*_6_ *f* complex which oxidizes plastiquinol (PQH_2_) to plastoquinone (PQ). CSK senses the redox state of the plastoquinone pool directly by becoming autophosphorylated and activated by PQ. CSK phosphorylation and dephosphorylation initiate transcription of PS II reaction centre (*psbA,D*) and PS I reaction centre (*psaA,B*) genes, respectively, selectively controlling expression of reaction centre genes in chloroplast DNA. The LHC II kinase Stn7 responds to PQH_2_ and initiates the state 2 transition, while the phospho-LHC II phosphatase, TAP38/PPH1, is redox-independent and predominates, inducing the state 1 transition, when PQ is oxidized. Even though they are both controlled by plastoquinone redox state, CSK exerts its transcriptional effect on photosystem stoichiometry independently of the effect of Stn7 in state transitions.

Since CSK is not involved directly in the mechanism of redox sensing in state 1- state 2 transitions (Figures 2 and 3), and a protein kinase known as Stt7/Stn7 has been implicated in state transitions by acting as the LHC II kinase, it seems necessary to assume that a bifurcated redox signalling pathway carries information from the plastoquinone pool, as depicted in Figure 4. One distal branch of the pathway, containing Stt7/Stn7, affects post-translational modification of existing proteins by phosphorylation. The second branch, consisting of CSK, controls photosystem stoichiometry by means of regulation of transcription of chloroplast genes for reaction centre apoproteins. Upstream of the point of divergence of the two branches is plastoquinone itself. It remains to be seen whether two separate plastoquinone/quinol-binding sensors initiate the two signal transduction events, or whether a single plastoquinone-binding redox sensor, as yet unidentified, controls both CSK and the LHC II kinase. The first possibility, that plastoquinone redox state is sensed by two independent redox sensors – CSK and LHC II kinase – is supported by the available evidence and consistent with a recent model for redox control of Stn7 [35].

It will also be important to resolve the evolutionary origin of these related redox signal transduction pathways. Plastoquinone itself is common to electron transport in both chloroplasts and cyanobacteria, and in both cases, both state transitions and photosystem stoichiometry appear to be initiated by changes plastoquinone redox state [9], [60], [61]. CSK and its homologues [62] are likely to be involved in transcriptional control in all cases, while the differing peripheral light-harvesting antenna systems of cyanobacteria and chloroplasts make the participation of an LHC II kinase and phosphatase in cyanobacterial state transition unlikely. Nevertheless, quinone-level redox control seems to be a conserved feature of regulation in a very wide range of bioenergetic systems and it is usual in prokaryotic signal transduction for a single environmental input to exert effects at multiple levels of gene expression, from transcription to post-translational modification of pre-existing proteins [63], [64], [65], [66].

## Materials and Methods

### Plant material

*Arabidopsis thaliana* (Col-0) obtained from Nottingham Arabidopsis Stock Centre (NASC) was grown from seed at 24°C with an 8-hour day at 120 µE m^−2^ s^−1^. The CSK T-DNA line (SALK_027360) was obtained from the Arabidopsis Biological Resource center (ABRC) and grown under the same conditions. The CSK T-DNA line used is one of the two well-characterised T-DNA insertion lines used in our earlier investigation of the *CSK* phenotype [47]. This T-DNA line has been genotyped for homozygous T-DNA insertion, and the absence of transcription from the *CSK* gene locus has been confirmed by a Reverse Transcriptase Polymerase Chain Reaction (RT-PCR), as previously described [47].

### Room temperature fluorescence measurement

State transitions were monitored by recording room temperature variable photosystem II fluorescence using a Walz PAM 101 chlorophyll fluorometer with the 101-ED emitter-detector unit. 6–8 week old plants, grown under white light, 120 µE m^−2^ s^−1^, 8-hour day, were used for the measurement. Before measuring state transitions, one set of plants were given two days of light 1 treatment to increase the size of the photosystem II antenna, while the other set of plants remained in white light condition. State transitions were thus measured for both light 1-treated and white light grown plants. Light 1, with a photon flux density of 6 µE m^−2^ s^−1^, was provided by two red fluorescent strip lamps (Osram L 18W/60 Red from Osram GmbH, Hellabrunner Straße 1, 81536 München Germany) each wrapped in red filter (Lee 027 medium red from Lee Filters, Andover, Hants, U.K.). For the Walz PAM measurements Light 1 was provided in to the fibre-optic bundle by the Walz 102-FR far-red photodiode array (maximum intensity at 735 nm) and light 2 was similarly provided by a Flexilux 150 HL white light source behind a Corning 4–96 glass filter. Maximal fluorescence (Fm) in state 1 and 2 were measured by a 0.8 second long, saturating (6500 µE m^−2^ s^−1^) light flash delivered from a Schott KL 1500 white light source.

### 77 K fluorescence measurement

Fluorescence emission spectra from wild type and CSK mutant thylakoids were recorded in liquid nitrogen (77 K) using the Perkin-Elmer LS 55 luminescence spectrometer. Thylakoid membranes were prepared as in Harrison and Allen [67]. State 1 was induced by 20 minutes of dark incubation of thylakoids in the presence of 0.4 mM final concentration of ATP and state 2, by illuminating the thylakoid suspension with 80 µE m^−2^ s^−1^ of white light for 20 minutes and by the addition of 0.4 mM final concentration of ATP. 20 minutes of dark incubation of thylakoids was done as a control treatment (results not shown). Thylakoid suspension equivalent to 5 µg/ml chlorophyll was taken per sample. The excitation wavelength was 435 nm (5 nm slit width) and emission was detected from 650 to 800 nm (2.5 nm slit width). All spectra were normalized at 685 nm.

### Thylakoid protein phosphorylation

*Arabidopsis* thylakoids were isolated from detached leaves of two-month old plants grown under white light, 120 µE m^−2^ s^−1^, 8-hour day. Isolation was as described by Harrison and Allen [67]. The thylakoid suspension was kept in the dark, on ice, for 45 minutes before the start of the experiment to ensure dephosphorylation. Incubation conditions used were light (80 µE m^−2^ s^−1^), light plus DCMU (at 10 µM final concentration) and dark plus sodium dithionite (at 20 mM final concentration). Reactions were initiated by the addition of ATP to thylakoid suspension equivalent to 16 µg of chlorophyll per reaction to give 0.4 mM final concentration of ATP with 10 µCi [*γ*-^32^P] ATP (3000 Ci/mmol). Reactions were stopped after 10 minutes by addition of electrophoresis sample buffer. Samples were loaded onto a 12–20% SDS-PAGE gel, with a constant loading of 16 µg chlorophyll per lane. The gel was stained with Coomassie brilliant blue, and exposed to a phosphor-imager plate for 36 hours.
